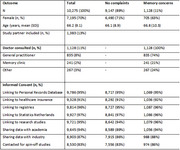# Towards a Dutch nationwide registry for Alzheimer's disease and other dementia's: rationale, design, and initial observations of the ABOARD Cohort

**DOI:** 10.1002/alz70860_098974

**Published:** 2025-12-23

**Authors:** Casper de Boer, Hanneke F.M. Rhodius‐ Meester, Sophie M. van der Landen, Jurgen A.H.R. Claassen, Janne M. Papma, Harro Seelaar, Marleen Kloppenburg‐Lagendijk, Barbara C. van Munster, Marjolein de Vugt, Derk Arts, Marco Blom, Tanja J. de Rijke, Miriam Beusink, Robbert Huijsman, Evert‐Ben van Veen, Argonde C. van Harten, Everard G.B. Vijverberg, Marissa D. Zwan, Henk Mutsaerts, Sven J van der Lee, Wiesje M. van der Flier

**Affiliations:** ^1^ Amsterdam Neuroscience, Neurodegeneration, Amsterdam, Netherlands; ^2^ Alzheimer Center Amsterdam, Neurology, Vrije Universiteit Amsterdam, Amsterdam UMC location VUmc, Amsterdam, Netherlands; ^3^ Department of Geriatric Medicine, The Memory Clinic, Oslo University Hospital, Oslo, Norway; ^4^ Alzheimer Center Amsterdam, Neurology, Amsterdam UMC Location VUmc, Vrije Universiteit Amsterdam, Amsterdam, Netherlands; ^5^ Department of Geriatrics & Radboudumc Alzheimer Center, Radboud University Medical Center, Nijmegen, Netherlands, Netherlands; ^6^ Alzheimer Center Erasmus MC, Rotterdam, South Holland, Netherlands; ^7^ Erasmus MC University Medical Center, Rotterdam, Rotterdam, Netherlands; ^8^ Medisch Centrum Leeuwarden, Leeuwarden, Netherlands; ^9^ University of Groningen, University Medical Center Groningen, Groningen, Netherlands; ^10^ Alzheimer Center Limburg, Maastricht University Medical Center+, Maastricht, Netherlands; ^11^ Castor EDC, Amsterdam, Netherlands; ^12^ Alzheimer's Netherlands, Amersfoort, Netherlands; ^13^ Medical Psychology, Amsterdam UMC location AMC, University of Amsterdam, Amsterdam, Netherlands; ^14^ Health‐RI, Utrecht, Utrecht, Netherlands; ^15^ Erasmus University Rotterdam, Rotterdam, Netherlands; ^16^ MedLaw consult, Voorburg, Netherlands; ^17^ Alzheimer Center Amsterdam, Department of Neurology, Amsterdam UMC, location VUmc, Amsterdam, Netherlands; ^18^ Amsterdam Neuroscience, Neurodegeneration, Amsterdam, Noord‐Holland, Netherlands; ^19^ Amsterdam Neuroscience, Brain Imaging the Netherlands, Amsterdam, Netherlands; ^20^ Amsterdam UMC, Amsterdam, Netherlands; ^21^ Section Genomics of Neurodegenerative Diseases and Aging, Department of Human Genetics, Vrije Universiteit Amsterdam, Amsterdam, Noord‐Holland, Netherlands; ^22^ Alzheimer Center Amsterdam, Amsterdam, Netherlands; ^23^ Department of Epidemiology and Data Science, Amsterdam UMC, Amsterdam, Netherlands

## Abstract

**Background:**

Alzheimer's disease (AD) takes 20 to 30 years to develop, yet hardly any existing data collection or registry takes into account the entire disease trajectory. Moreover, prediction models are often based on selected research populations and their outcomes may not be most relevant to patients’ daily lives. To address these gaps we set up a Dutch national data collection infrastructure, the ABOARD Cohort, to (i) study the AD disease trajectory using patient reported outcome measures (PROMs) and medical data, (ii) link to available registry data, and (iii) serve as central platform to initiate additional studies and roll‐out healthcare innovations. Here, we describe the design of the project and characteristics of the first 10,275 participants.

**Method:**

The ABOARD Cohort is an ongoing, participant‐centered data‐collection, focused on PROMs, a minimal case report form (CRF) with relevant medical data, and linkage to existing data sources (Figure 1). Eligible participants with or at‐risk of AD or other types of dementia and their study partners are recruited directly‐to‐participant. Informed consent and annual collection of PROMs are fully online. Relevant stakeholders are involved in decisions on project development through a panel of participants and on data usage through a data access committee.

**Results:**

The ABOARD Cohort has been operational since January 2023. As of October 2024, 10,275 participants (mean age 66.1 ± 9.2 years, 70% female) and 1,383 partners signed up, and received an invitation to fill in online questionnaires and complete a digital cognitive test (table 1). Over 90% of those participants also gave consent to link their data to existing registries. Participants who had consulted a doctor for memory problems (*N* = 1,128), reported worse outcomes on PROMs assessing mental health and cognition, quality of life and lifestyle, compared to those who had not.

**Conclusion:**

The ABOARD Cohort has been set up as a national infrastructure to study AD disease trajectories, linking data‐sources, with the participant at the steering wheel. This infrastructure has the potential to serve as a future registry to advance AD research on a national level, and provide Real World Data to evaluate novel interventions and therapies.